# Timing Intervals and Frequency of Adenoma Surveillance Colonoscopies in Central Queensland, Australia

**DOI:** 10.1111/ans.70517

**Published:** 2026-03-02

**Authors:** Yiu Ming Ho, Katharina M. D. Merollini, Louisa G. Collins

**Affiliations:** ^1^ Department of Surgery The Prince Charles Hospital Chermside Queensland Australia; ^2^ School of Medicine The University of Queensland Herston Queensland Australia; ^3^ School of Health University of the Sunshine Coast Sippy Downs Queensland Australia; ^4^ Sunshine Coast Health Institute Sunshine Coast University Hospital Birtinya Queensland Australia; ^5^ Cancer Research Cluster University of the Sunshine Coast Sippy Downs Queensland Australia; ^6^ Viertel Cancer Research Centre, Cancer Council Queensland Queensland Australia; ^7^ School of Public Health The University of Queensland St. Lucia Queensland Australia; ^8^ School of Nursing The Queensland University of Technology Kelvin Grove Queensland Australia

## Abstract

**Introduction:**

The presence of colonic adenomatous polyps is a risk factor for colorectal cancer. Australian Guidelines changed in 2019 so that the surveillance colonoscopy intervals and polyp risk stratification were changed to reflect evidence available. The purpose of this research was to understand compliance with the Guidelines and implications for the health system.

**Methods:**

Using routine hospital administrative datasets, all polyp surveillance colonoscopies performed from January 2018 to September 2020 in three government‐funded hospitals in Central Queensland, Australia, were analysed. Colonoscopy intervals were calculated and compared with national recommendations. ‘Early surveillance’ was defined as greater than 6 months earlier than recommended. Logistic regression analyses were used to assess early surveillance or not, adjusted for potential confounding. Interval cancer and adenoma detection rates were also examined.

**Results:**

Surveillance colonoscopies were performed for 294 patients with low‐risk polyps, 20 with intermediate‐risk polyps, 321 with high‐risk polyps and 12 with very high‐risk polyps during the study period (total *n* = 647). Early surveillance occurred in 566 (87.5%). The overall interval cancer rate was 0.9% (6/647), and adenoma detection rates were 62.2% (357/574) before the change of guidelines and 79.1% (53/67) after the change. No examined demographic or clinical factors were associated with early surveillance.

**Conclusion:**

Despite outstanding and high‐quality colonoscopy services being provided, higher than recommended colonoscopy surveillance was identified in the regional public hospitals in Central Queensland. Hospital processes should be improved to ensure appropriate intervals between procedures to avoid using scarce healthcare resources.

## 1 Introduction

Significant colonic polyps are a recognised risk factor for colorectal cancers [1]. Surveillance colonoscopies at intervals guided by initial colonoscopy findings are recommended for patients with significant polyps, mainly adenoma and sessile serrated adenoma (SSA), detected in an index colonoscopy, by the National Health and Medical Research Council (NHMRC) of Australia guidelines: *Surveillance colonoscopy Clinical practice guidelines for surveillance colonoscopy* [2]. These guidelines have improved over time since 2005, supported by emerging clinical evidence, and are consensus‐based with an expert working group organised through Cancer Council Australia [2].

Polyp surveillance is an essential component of comprehensive colonoscopy services and accounts for up to 25% of total colonoscopies performed [3]. There is evidence that increasing the frequency of colonoscopies does not necessarily result in a reduction in colorectal cancer‐related mortality rates [4]. National Guidelines emphasise the importance of considering polyp size and pathological features in determining surveillance intervals [5]. To optimise the use of colonoscopy resources while minimising the risk of over‐servicing, risk stratification approaches have been adopted [6]. Nevertheless, over‐servicing is a common issue, and a recent systematic review showed that globally, up to 40% of polyp surveillance colonoscopies are performed earlier than country‐specific recommendations [7].

Australian Guidelines stratify colorectal cancer risk of patients according to the polyps found during their colonoscopies. In the pre‐2019 Guidelines [5], patients with one to two tubular adenomas which measure less than 10 mm with low grade dysplasia were stratified as low risk. The recommended colonoscopy interval from the Guidelines was 5 years. Patients with two or more adenomas with at least one adenoma larger than 10 mm, or with high‐grade dysplasia, were instructed to have their next colonoscopy at 3 years. Following a change of the Guidelines in November 2019, intervals were now risk‐stratified based on up‐to‐date evidence on disease‐specific risk from colonic polyps [2] (see Table A1). The purpose of this study was to evaluate adherence to the pre and post 2019 polyp surveillance Guidelines and identify potential areas for improvement.

## 2 Methods

### 2.1 Study Design

This is a retrospective, observational study. We adhered to the Strengthening the Reporting of Observational studies in Epidemiology (STROBE) statement for reporting observational studies [8]. The study was approved by the Human Research Ethics Committee of Central Queensland Hospital and Health Service (CQHHS) in 2021 (HREC/2021/QCQ69478).

### 2.2 Setting

Central Queensland is in regional Queensland, Australia and has a total population of 228 000 [9]. Rockhampton Hospital, Gladstone Hospital and Emerald Hospitals are the three government hospitals providing colonoscopy services in the Central Queensland health district [10].

### 2.3 Participants

Adults who received polyp surveillance colonoscopies from the 1st of January 2018 to the 30th of September 2020 in three government hospitals in Central Queensland were selected for the analyses. Surveillance colonoscopy was defined as colonoscopy performed with ‘Polyp follow up’ or ‘polyp surveillance’ as the documented indication on the colonoscopy report. Cases were excluded if the indication for the colonoscopy performed was unclear after verification with the principal investigator (YMH).

### 2.4 Demographic and Clinical Variables

Socio‐demographic information was collected, including the year of birth, sex, marital status and Aboriginal and Torres Strait Islander status. Clinical service details, including colonoscopy date, pathology results and details of any complications were collected. Findings during the colonoscopy, such as the presence and removal of colonic adenoma, were extracted. Information on polyp removal, including number, size and the presence of advanced features, impacts surveillance intervals and were collected. Histology reports were reviewed to confirm the presence and the details of the adenomata removed. Colonoscopies with piecemeal polypectomy have a different surveillance regimen and often had sigmoidoscopy surveillance and were therefore excluded. Factors that might affect colonoscopy intervals, including American Society of Anaesthesiologists (ASA) score and the quality of bowel preparation, were collected. The ASA score is a standardised system used to assess a patient's overall physical health and clinical risk [11].

### 2.5 Measurement of Guidelines Adherence

Staff underwent training on the risk categorisation framework and were familiarised with the Guidelines prior to undertaking data collection. Queries arising during the data collection process were addressed through consultation with the principal investigator (YMH). We used different versions of the guidelines [5, 12], either before or after the update in November 2019, to ensure comprehensive analysis of the data. The index colonoscopy was denoted by C1. The subsequent surveillance colonoscopy (C2) was early if the interval was less than 4.5 years using a grace period of 6 months, to allow time for scheduling appointments and waiting list time. Similarly, the surveillance interval was considered late if it was more than 5.5 years. This period is arbitrary but has been used previously by others [7]. High risk patients should have a surveillance interval of 3 years and were regarded as early if performed less than 2.5 years and late if it occurred after more than 3.5 years.

Colonoscopy intervals were compared against the Guidelines and categorised for ‘early’, ‘appropriate’ and ‘late’. After the Guidelines‐change in November 2019, for low risk patients, the interval should be 10 years and was regarded as early if less than 9.5 years. For intermediate risk patients, 5‐year interval was appropriate, early if less than 4.5 years and late if more than 5.5 years. For high risk patients, 3‐year interval was appropriate, early if less than 3.5 years and late if more than 2.5 years. For very high‐risk patients, 1‐year interval was appropriate, early if less than 6 months and late if more than 1.5 years (see Table A2). In these updated Guidelines, the results from the two prior colonoscopies also affects the risk stratification and such information was collected in the study.

### 2.6 Outcome Reporting

Colonoscopy intervals were reported in days. Colonoscopy findings included polyp details, adenoma detection rate (ADRs) and SSA rates, interval cancer rates, complication rates and quality of bowel preparation. The perforation rate was defined as the number of perforation events per surveillance colonoscopy performed. Perforation events were identified through review of the electronic medical records.

### 2.7 Statistical Methods

Summary statistics were used to describe socio‐demographic, clinical and colonoscopy timing characteristics. Continuous variables were summarised and presented as means and standard deviations (SD) while categorical variables were presented as counts and percentages. Binary logistic regression analyses were conducted separately for patients to identify any socio‐demographic factors associated with patients having early surveillance, compared with either on time or late surveillance. Factors considered in the regression analysis were age at the resection, sex, marital status, ASA and quality of bowel preparation. Statistical significance was defined by *p* < 0.05. Missing socio‐demographic or clinical data were reported in the results. Analyses were performed using STATA (version 16.1, Texas, USA).

## 3 Results

A total of 2814 patients were identified as having undergone colonoscopy during the study period across three sites in Central Queensland. Of these, 647 patients (23.0%) underwent colonoscopy for polyp surveillance and were included in this analysis. Three patients were excluded due to missing data, and one data point was excluded due to duplication. The mean age at the time of resection was 70 years (SD 10.8; interquartile range [IQR] 16) (see Table 1 for demographic details). The quality of bowel preparation was rated as excellent or good in the majority of surveillance colonoscopies (237, 41%, in C1 and 330, 57%, in C2). However, documentation of bowel preparation quality was frequently missing (333, 58%, in C1 and 201, 35%, in C2).

**TABLE 1:** Cohort demographics.

	Number of colonoscopy intervals (index colonoscopy performed prior to November 2019)		Number of colonoscopy intervals (index colonoscopy performed in or after November 2019)		Total (*n*)	%
	*n*	%	*n*	%		
	579	89.5%	68	10.5%	647	100%
Age at C1						
Mean (SD)	70.3 (10.7)		67.6 (11.4)		70.0 (10.8)	
Median (IQR)	72 (15)		68 (19.25)		72 (16)	
< 50 years	21	91.3%	2	8.7%	23	100%
50–59	70	80.5%	17	19.5%	87	100%
60–69	159	88.8%	20	11.2%	179	100%
70–79	207	93.2%	15	6.8%	222	100%
80+	122	89.7%	14	10.3%	136	100%
Sex						
Female	192	89.3%	23	10.7%	215	100%
Male	387	89.6%	45	10.4%	432	100%
ASA score						
1	33	91.7%	3	8.3%	36	100%
2	307	90.0%	34	10.0%	341	100%
3	210	87.9%	29	12.1%	239	100%
4	11	84.6%	2	15.4%	13	100%
Not documented	18	100.0%	0	0.0%	18	100%
Marital status						
Married or de facto	301	89.6%	35	10.4%	336	100%
Never married	109	87.9%	15	12.1%	124	100%
Other	168	90.3%	18	9.7%	186	100%
Not documented	1	100.0%	0	0.0%	1	100%
ATSI status						
Neither	557	89.2%	67	10.8%	624	100%
ATSI	21	95.5%	1	0.5%	22	100%
Not documented	1	100.0%	0	0.0%	1	100%

Abbreviations: ASA, American Society of Anesthesiologists; ATSI, Aboriginal and Torres Strait Islander; IQR, interquartile range; SD: standard deviations.

### 3.1 Proportion of Early Surveillance Colonoscopy

**FIGURE 1:**
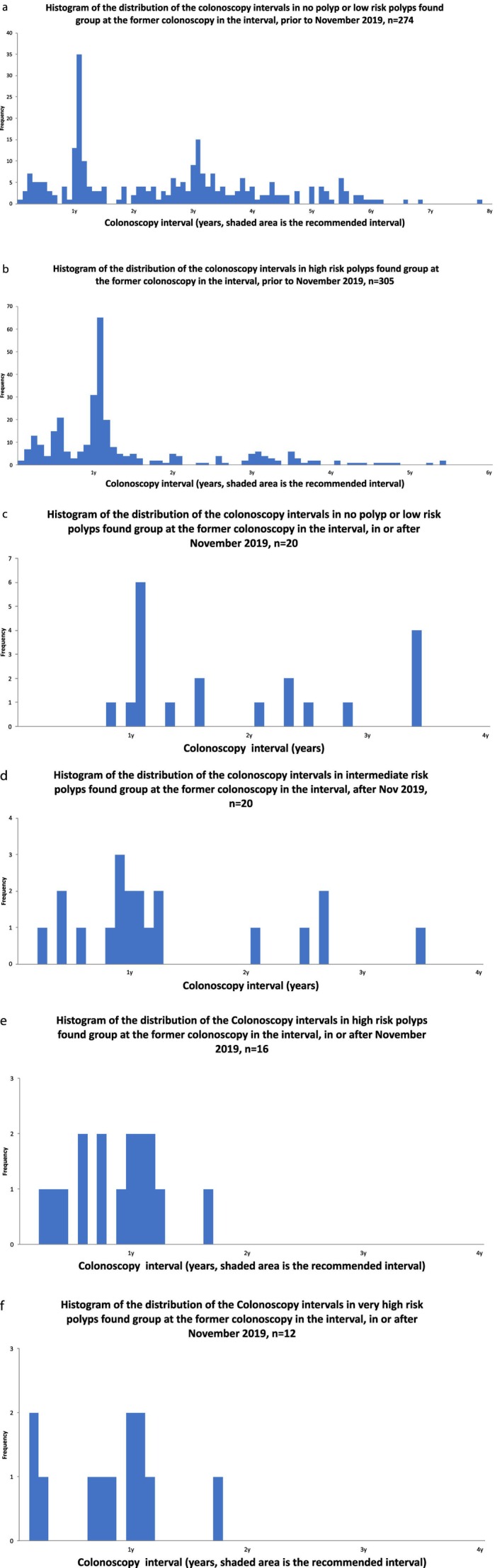
(a–f) Histograms of the distribution of the colonoscopy intervals in different risk groups at the former colonoscopy in the interval, across the guidelines change in November 2019; shaded area indicates the guideline recommended surveillance interval for the polyps, with a grace period of 6 months.

Among 579 patients (89.4% of 647) who underwent their first colonoscopy prior to the change in Guidelines, 274 (47.3%) had low‐risk polyps or no adenoma, of whom 255 (93.1%) received early surveillance. Among the 305 patients (52.7%) with high‐risk polyps, 252 (82.6%) underwent early surveillance colonoscopy. Overall, including those not requiring surveillance, 507 patients (87.6%) received surveillance colonoscopies early (see Table 2 for detailed colonoscopy intervals by polyps' risk and Figures 1a–f for graphical representations of colonoscopy intervals). The mean surveillance interval was 634 days (SD 505.2), with a median of 505 days (range: 14–2852 days). The mean and median differences between actual and recommended intervals were −562 days and −548.5 days, respectively (SD 509.6; IQR 405.75), indicating surveillance occurred earlier than recommended.

**TABLE 2:** Colonoscopy intervals.

	Number of colonoscopy intervals (index colonoscopy performed prior to November 2019)		Number of colonoscopy intervals (index colonoscopy performed in or after November 2019)		Total number of colonoscopy intervals	
C1: No polyp or low‐risk group						
CIs Compare against the Guidelines						
Early	255	92.7%	20	7.3%	275	100%
Appropriate	4	100.0%	0	0.0%	4	100%
Late	15	100.0%	0	0.0%	15	100%
C1: intermediate‐risk group						
CIs compare against the guidelines						
Early	N/A	N/A	20	N/A		
Appropriate	N/A	N/A	0	N/A		
Late	N/A	N/A	0	N/A		
C1: high‐risk group						
CIs compare against the guidelines						
Early	252	94.0%	16	6.0%	268	100%
Appropriate	12	100.0%	0	0.0%	12	100%
Late	41	100.0%	0	0.0%	41	100%
C1: very high‐risk group						
CIs compare against the guidelines						
Early	N/A	N/A	3	N/A		
Appropriate	N/A	N/A	8	N/A		
Late	N/A	N/A	1	N/A		
Overall CIs						
CIs compare against the guidelines						
Early	507	89.6%	59	10.4%	566	100%
Appropriate	16	66.7%	8	33.3%	24	100%
Late	56	98.2%	1	1.8%	57	100%
Overall CIs (%)		%		%		
CIs compare against the guidelines						
Early		87.6%		86.8%		
Appropriate		2.8%		11.8%		
Late		9.7%		1.5%		

Abbreviations: C1, the former colonoscopy in a colonoscopy interval; CIs, colonoscopy intervals.

### 3.2 Factors Associated With Early Surveillance

No significant associations were found between early surveillance and sex, ATSI status, marital status, patient age at the first colonoscopy, quality of bowel preparation at the first colonoscopy or ASA score at the first colonoscopy (Table 3).

**TABLE 3:** Binomial logistic regression analysis of factors that contributed to early surveillance.

Variables	Crude		Adjusted	
	OR	95% CI	OR	95% CI
Age	1.01	0.99–1.03	1	0.98–1.02
Sex				
Male	1		1	
Female	1.06	0.64–1.75	1.12	0.67–1.90
Marital status				
Married or de facto	1		1	
Never married	1.17	0.69–1.98	1.26	0.73–2.17
Other	1.28	0.63–2.55	1.51	0.72–3.15
Not documented	1 (omitted)		1 (omitted)	
ASA				
1	1		1	
2	0.83	0.19–3.67	0.77	0.16–3.66
3	1.35	0.38–4.85	1.25	0.34–4.66
4	1.57	0.43–5.78	1.44	0.37–5.62
ATSI				
No	1		1	
Yes	2.12	0.76–5.91	1.85	0.62–5.46
Bowel prep quality				
Excellent	1		1	
Good	1.54	0.88–2.71	1.60	0.89–2.88
Adequate	2.20	0.76–6.36	2.23	0.76–6.54
Inadequate	0.43	0.08–2.29	0.46	0.08–2.56
Poor	1.12	0.25–5.12	1.21	0.26–5.66
2019 guideline change				
Before 2019	1		1	
After 2019	0.93	0.44–1.96	0.8	0.37–1.74

Abbreviations: ASA, American Society of Anaesthesiologists; ATSI, Aboriginal and Torres Strait Islander; CI, confidence interval; OR, odds ratio.

### 3.3 Secondary Outcomes

There were six interval cancers (0.9% of 647) detected in the study period. There was no statistically significant association between early surveillance and interval cancer detection (*p* = 0.255). Similarly, no association was found between early surveillance and ADR (*p* = 0.255). The ADR was 59.2% for appropriately timed surveillance, 62.0% for early surveillance, 56.1% for late surveillance, and 49.1% for out‐of‐indication surveillance (see Table 4 for detailed colonoscopy findings).

**TABLE 4:** Colonoscopy findings.

	Number of colonoscopy intervals (index colonoscopy performed prior to November 2019)		Number of colonoscopy intervals (index colonoscopy performed in or after November 2019)		Total (*n*)	%
	*n*	%	*n*	%		
C1 findings						
C1: No adenoma	81	90.0%	9	10.0%	90	100%
C1: Has adenoma	498	89.4%	59	10.6%	557	100%
C1: Mean (SD) number of adenomas	2.4 (2.1)		3.3 (3.3)			
C1: ADR	86.0%		86.8%			
C1: adenoma with no adverse features	314	91.0%	31	9.0%	345	100%
C1: adenoma with adverse features	184	86.8%	28	13.2%	212	100%
C1: has SSA	108	87.8%	15	12.2%	123	100%
C1: SSA rate	18.7%		22.1%			
C1: cancer detected	0		0		0	
C1 risk stratification from polyp findings						
No adenoma	81	93.1%	9	6.9%	87	100%
Low risk	193	93.2%	14	6.8%	207	100%
Intermediate risk	—	—	20	—	—	—
High risk	305	95.0%	16	5.0%	321	100%
Very high risk	—	—	12	—	—	—
C2 findings						
C2: No adenoma	217	93.9%	14	6.1%	231	100%
C2: Has adenoma	357	87.1%	53	12.9%	410	100%
C2: Mean (SD) number of adenomas	1.6 (2.2)		2.3 (2.3)			
C2: ADR	62.2%		79.1%			
C2: adenoma with no adverse features	280	84.9%	50	15.1%	330	100%
C2: adenoma with adverse features	77	96.3%	3	3.7%	80	100%
C2: has SSA	79	85.0%	14	15.0%	93	100%
C2: SSA rate	13.6%		20.6%			
C2 cancer/advanced polyp requiring surgery detected	5	83.3%	1	16.7%	6	100%
Interval cancer rate	0.9%		1.5%			

Abbreviations: ADR, adenoma detection rate; C1, the former colonoscopy in a colonoscopy interval; C2, the latter colonoscopy in a colonoscopy interval; IQR, interquartile range; SD, standard deviations; SSA, sessile serrated adenoma.

### 3.4 Adverse Events

Three adverse events (0.4%) were recorded. Two involved oxygen desaturation, and one was a suspected aspiration, which required admission for further management. There were two perforations (0.07% of 2814) observed in the entire cohort, but none occurred for patients with an indication of polyp surveillance.

## 4 Discussion

Our study found that public hospital Australian patients received polyp surveillance colonoscopies far more frequently than recommended by risk‐based clinical guidelines. There were no obvious socio‐demographic or clinical features explaining early surveillance colonoscopies. The high proportion of early surveillance colonoscopy observed in this study was consistent with a previous systematic review and meta‐analysis [7]. This may reflect persistent clinician and patient concerns about missed or recurrent lesions, despite updated Guidelines recommending longer surveillance intervals. This cautionary and ongoing practice may be driven by historical practices, limited awareness or acceptance of newer recommendations and medico‐legal considerations [13]. The principal investigator was one of the endoscopists at the CQHHS during the study period. No financial drive to increase surveillance, either at a staff or hospital administration level, was encountered. At an institutional level, the presence of a substantial colonoscopy waitlist makes surveillance colonoscopy practises within the HHS influenced by financial incentives less plausible, as demand for colonoscopy services already exceeds capacity. Furthermore, patients whose wait times exceed recommended thresholds incur financial penalties to the HHS under health service arrangements, which would act as a disincentive to unnecessary procedural volume.

On a national level, this study corroborates the widespread practice of early polyp surveillance [14]. However, the increasing demand for colonoscopy services places additional strain on already overburdened healthcare systems, particularly in regional areas. The *Atlas Focus Report* from the Australian Commission on Safety and Quality in Health Care (ACSQHC) 2025 highlights significant disparities in colonoscopy access and timing across different socioeconomic and geographic regions [15]. Central Queensland is one regional region facing limited access to colonoscopy services. This issue of access further exacerbates challenges related to the Australian National Bowel Cancer Screening Program (NBCSP), as noted by Worthington et al. [16]. Encouraging participation in NBCSP is an important strategy to reduce the mortality of colorectal cancer. Timely access to colonoscopy is a critical subsequent step for individuals with a positive faecal occult blood test. However, access may be impeded by over‐servicing and potential bottlenecks.

The lack of a significant association between early surveillance and improved clinical outcomes, specifically interval cancer or adenoma detection rates, suggests that early surveillance may not provide additional benefit in appropriately risk‐stratified patients [17]. Indeed, the ADR was comparable across early (62.0%), appropriate (59.2%) and late (56.1%) surveillance groups. Notably, the lowest ADR (49.1%) was observed in patients who underwent surveillance without prior adenoma detection, raising concerns about overuse of hospital resources in low‐risk populations. Interestingly, the subgroup of polyp surveillance without prior adenoma detection still demonstrated an ADR of nearly 50%, which, while considerably higher than the Gastroenterological Society of Australia benchmark, may reflect procedural quality more than patient risk. This suggests some polyps detected in these ‘out‐of‐indication’ procedures may be clinically insignificant, supporting the concept of non‐progressing or indolent adenomas [18]. We do not advocate for leaving polyps unresected during colonoscopy; rather, the results in this study supported the notion that a colonoscopy with findings of a low‐risk profile is associated with a very low risk of disease‐specific mortality. Consequently, surveillance colonoscopy in this clinical context is likely to confer little, if any, additional benefit to the individual in terms of CRC prevention. The excessive polyp surveillance colonoscopy may highlight potential over‐servicing, possibly driven by routine direct bookings for surveillance colonoscopy post‐polypectomy without histological confirmation.

The broadening of data collection and analysis related to polyp surveillance for future research, particularly in private healthcare settings and across both private and public facilities, presents significant challenges. Nevertheless, such data collection is critical for accurately assessing the extent of over‐servicing in this area and for informing future policy and practise adjustments.

## 5 Limitations and Strengths

Several limitations warrant careful interpretation of the study results. Firstly, the retrospective design and regional public setting may limit generalisability to other healthcare contexts, including urban centres or private practise. Additionally, the assessment of bowel preparation quality lacked standardisation. Objective grading tools such as the Boston Bowel Preparation Scale [19] were not routinely used and more than half of the preparation qualities were not documented, which may have limited the accuracy of that analysis. A key strength of this study was the full capture of all cases in the Central Queensland district, largely due to the compulsory reporting of cancer cases, over an extended follow‐up period. This allowed for comprehensive capture of cancer‐related treatment activities from a bottom‐up perspective.

## 6 Conclusion

The polyp surveillance colonoscopy service provided by CQHHS was delivered safely and to a high standard. However, a significant proportion of patients receiving colonoscopies for polyp surveillance are unnecessarily performed too frequently.

## Funding

This work was supported by the Royal Australasian College of Surgeons.

## Data Availability

The data that support the findings of this study are available from the corresponding author upon reasonable request.

## Appendix A

**TABLE A1:** Risk stratification based on polyp number and features across guideline‐recommended surveillance intervals.

Pre November 2019‐guideline	Definition of risk	Recommended surveillance	Post November 2019‐guideline	Definition of low‐risk	Recommended surveillance
Low risk	One to two tubular adenomas < 10 mm with low grade dysplasia	5 years	Low risk	One to two tubular adenomas < 10 mm with low grade dysplasia	10 years or RTS
Intermediate risk	n/a	n/a	Intermediate risk	Three to four adenomas without villous features, high‐grade dysplasia, and less than 10 mm One to two adenomas with villous features, high‐grade dysplasia, or larger than 10 mm; or One to two SSAs without high‐grade dysplasia and less than 10 mm	5 years
High risk	Two or more adenomas with at least one adenoma larger than 10 mm, or high‐grade dysplasia	3 years		Five to nine adenomas without villous features, or high‐grade dysplasia, and less than 10 mm One to two adenomas with villous features, high‐grade dysplasia, or larger than 10 mm Three to four adenomas without villous features, high‐grade dysplasia, but larger than 10 mm One to two SSAs with high‐grade dysplasia or larger than 10 mm Three to four SSAs without high‐grade dysplasia and less than 10 mm	3 years
Very high risk	n/a	n/a		Five to nine adenomas with villous features, high‐grade dysplasia, but less than 10 mm Five to nine adenomas larger than 10 mm; or more than 10 adenomas Three to four SSAs with high‐grade dysplasia or less than 10 mm More than five SSAs	

Abbreviations: RTS, return to screening; SSA, sessile serrated adenoma.

**TABLE A2:** A table summary of the time interval used in this study.

After the guidelines‐change	Early surveillance	Recommended surveillance	Late surveillance	After the guidelines‐change	Early surveillance	Recommended surveillance	Late surveillance
Low risk	Less than 4.5 years	5 years (4.5–5.5 years)	More than 4.5 years	Low risk	Less than 9.5 years	10 years (9.5–10.5 years)	More than 10.5 years
Intermediate risk	n/a	n/a	n/a	Intermediate risk	Less than 4.5 years	5 years (4.5–5.5 years)	More than 4.5 years
High risk	Less than 2.5 years	3 years (2.5–3.5 years)	More than 3.5 years	High risk	Less than 2.5 years	3 years (2.5–3.5 years)	More than 3.5 years
Very high risk	n/a	n/a	n/a	Very high risk	Less than 0.5 years	1 years (0.5–1.5 years)	More than 1.5 years
